# Exploring the Anti-Inflammatory Potential of a Mediterranean-Style Ketogenic Diet in Women with Lipedema

**DOI:** 10.3390/nu17183014

**Published:** 2025-09-20

**Authors:** Małgorzata Jeziorek, Angelika Chachaj, Andrzej Szuba, Dorota Różańska, Anna Prescha

**Affiliations:** 1 Department of Dietetics and Bromatology, Faculty of Pharmacy, Wroclaw Medical University, 50-556 Wroclaw, Poland; dorota.rozanska@umw.edu.pl (D.R.); anna.prescha@umw.edu.pl (A.P.); 2 Department of Angiology and Internal Medicine, Wroclaw Medical University, 50-556 Wroclaw, Poland; angelika.chachaj@umw.edu.pl (A.C.); andrzej.szuba@umw.edu.pl (A.S.)

**Keywords:** lipedema, women, ketogenic diet, Dietary Inflammatory Index (DII), systemic inflammation, anti-inflammatory diet

## Abstract

**Background/Objectives:** Lipedema is a chronic adipose tissue disorder characterized by disproportionate fat accumulation and inflammation, predominantly affecting women. While recent evidence suggests a systemic pro-inflammatory state in lipedema, the role of diet in modulating inflammation remains underexplored. This study assessed the anti-inflammatory potential of a Mediterranean-style ketogenic diet and its effects after 7 months of adherence on systemic inflammation markers (CRP and IL-6) in women with lipedema (n = 24) and a control group with overweight/obesity (n = 24). **Methods:** The Dietary Inflammatory Index (DII) was used to characterize the inflammatory potential of the diet throughout the intervention. Dietary intake was analyzed pre- and post-intervention, and anthropometric, body composition, and biochemical parameters were measured. **Results:** Beyond its beneficial effects on body composition (significant reductions in body weight, fat, leg circumferences, and visceral fat), the intervention diet also demonstrated anti-inflammatory potential. In lipedema, baseline diet showed a pro-inflammatory DII profile (DII/day = 3.04), which was reduced by about 1.5 points after the intervention (*p* = 0.008). When expressed per 1000 kcal, the DII values were markedly lower for both baseline (DII = 0.22) and intervention diet (DII = ~0.01). Following the intervention diet, reduction in CRP (−0.39, *p* = 0.016) and IL-6 levels (−0.33, *p* = 0.034) in lipedema were observed. A significant positive association was observed between the intervention diet’s DII and CRP (r = 0.55, *p* = 0.005), and between the baseline diet’s DII and IL-6 (r = 0.50, *p* = 0.013) in lipedema group. **Conclusions:** These findings suggest that ketogenic diet rich in anti-inflammatory and antioxidant nutrients can reduce systemic inflammation in lipedema patients, independently of caloric restriction.

## 1. Introduction

Lipedema is a condition characterized by the overgrowth of adipose tissue, particularly in the hips, thighs, and calves [1]. The disease predominantly affects women, with the prevalence in women estimated at approximately 11% [2,3]. The onset of lipedema most commonly follows major hormonal changes, such as puberty, pregnancy, and menopause. Although no single hormone has been definitively identified as the cause, estrogen-related pathways are the most strongly implicated. The underlying pathophysiological mechanism is thought to involve abnormal expression of estrogen receptors within adipose tissue [2]. However, it may not reflect the true incidence of the disorder, as diagnosing lipedema is challenging due to the variability of its symptoms [1]. The symptoms of lipedema include increased accumulation of pathologically altered adipose tissue, the formation of nodules within the subcutaneous layer that contribute to skin surface irregularities, and heightened tenderness or pain in the affected areas [1,4,5,6]. These symptoms significantly impact women’s quality of life, especially their self-acceptance and social openness [1,2].

Lipedema is increasingly recognized as an inflammatory disease, primarily due to a chronic, low-grade pro-inflammatory state in adipose tissue of the extremities [7,8]. However, systemic inflammation in lipedema remains less explored. While elevated circulating inflammatory markers may indicate widespread alterations in adipose tissue function, the development and clinical presentation of lipedema likely extend beyond adipose tissue involvement, as evidenced by additional symptoms such as pain in the extremities [8]. Recent studies have reported elevated serum concentrations of inflammatory markers such as CRP (C-reactive protein) and TNF-α (tumor necrosis factor-alpha) in lipedema patients [9,10]. In the study conducted by Nankam et al. [8] they identified significantly higher level of circulating inflammatory proteins (IL-11, IL-28a, and IL-19) in individuals with lipedema compared to obese controls, supporting the presence of a pronounced systemic pro-inflammatory signature in lipedema that exceeds the well-established low-grade inflammation seen in obesity. Al-Ghadban et al. [3] also reported that inflammation in lipedema occurs independently of obesity-related inflammation.

In an effort to reduce fat tissue, patients often implement lifestyle changes, including increased physical activity and low-energy diets. However, these strategies are generally ineffective, prompting the search for alternative solutions, including nutritional modifications aimed not only at adipose tissue reduction but also at exerting anti-inflammatory and anti-edematous effects [11]. One potential approach is the adoption of a ketogenic diet which primarily involves reducing the carbohydrate content and increasing the fat content of the diet. In our previous studies, we demonstrated that the use of a proprietary low-carbohydrate, high-fat diet, combined with a high intake of anti-inflammatory compounds, led to weight loss, reduction in adipose tissue content, decreased circumferences of the extremities and pain in patients with lipedema [12,13]. The ketogenic diet has been recognized for its effectiveness in producing fat loss, including a reduction in adipocyte size among lipedema patients. Although the diet is high in fat, its metabolic and anti-inflammatory effects provide a theoretical basis for potential benefit in lipedema. Specifically, the ketogenic diet induces nutritional ketosis, shifting energy metabolism from glucose to ketone bodies. This metabolic state has been shown to reduce both systemic and local inflammation, a key component of lipedema pathophysiology. In addition, it may promote mobilization of fat stores while preserving lean mass, thereby addressing weight management challenges commonly observed in lipedema. The ketogenic diet also exerts anti-edema effects, which may help alleviate pain and swelling [7]. A ketogenic diet, due to its high fat content and low in plant-based foods—which are sources of carbohydrates but also antioxidants—may increase inflammation [14,15]. However, if a high-fat diet may also provide large amounts of substances with anti-inflammatory and antioxidant properties, it may help counteract this risk. Moreover, the inflammatory effect of dietary fat depends on fat composition, as polyunsaturated fatty acids (PUFAs), particularly omega-3 fatty acids, as well as MUFA (monounsaturated fatty acids), have been shown to exert anti-inflammatory effects [7,16,17]. Recent studies found that ketogenic diet may lower levels of systemic inflammatory markers (TNF-α and CRP) in patients with lipedema [9,18]. Although, ketogenic diet has been proposed as a means to reduce inflammation in lipedema, the underlying mechanisms are not yet fully understood [7,9]. Given the growing interest in how specific diets may modulate inflammation, tools such as the Dietary Inflammatory Index (DII) have been developed to quantify the inflammatory potential of the diet. The DII has been validated against systemic inflammatory markers, making it a relevant instrument in exploring the diet-inflammation link [19,20]. The DII was developed through an extensive review and scoring of peer-reviewed literature, quantifying the effects of various dietary components on six key inflammatory biomarkers: interleukin-1β (IL-1β), IL-4, IL-6, IL-10, TNF-α, and CRP. Each dietary parameter was assigned a continuous score representing its pro- or anti-inflammatory potential, calculated from the weighted effects reported across studies [21]. Although the link between inflammatory markers and chronic disease outcomes is well established, the connection between DII and intermediate biomarkers of inflammation has not been investigated in patients with lipedema, particularly following a ketogenic diet.

The aim of the study was to evaluate the anti-inflammatory potential of the Mediterranean-style ketogenic diet using the DII and to analyze changes in systemic inflammation markers following a 7-month intervention diet in the lipedema and overweight/obesity groups.

## 2. Materials and Methods

### 2.1. Study Design and Study Groups

This was a 7-month prospective interventional study involving women with lipedema (n = 24) and a control group with overweight/obesity without lipedema (n = 24). All patients were referred from the Angiology Outpatient Clinic of Wroclaw Medical University. Inclusion criteria were a diagnosis of lipedema by an angiologist for the lipedema group, or a BMI > 25 kg/m^2^ with no lipedema symptoms for the control group, and age ≥ 18 years for both groups. The exclusion criteria for the study were: pregnancy, breastfeeding, the period up to 6 months postpartum, diagnosis of lymphedema, edema due to chronic venous insufficiency or heart failure, diabetes, renal or hepatic insufficiency, uncontrolled thyroid disease, cancer, implanted cardiac devices (pacemaker, implantable cardioverter-defibrillator, cardiac resynchronization therapy), or metal implants.

The study was initiated in January 2021 and concluded in May 2022. All participants provided written informed consent to take part in the study. The study was conducted in compliance with the Declaration of Helsinki, and all procedures involving human subjects were approved by the Bioethics Committee guidelines at Wroclaw Medical University, Poland (KB—690/2017) on 23 November 2017.

### 2.2. Body Composition and Anthropometric Parameters Measurements

A TANITA MC-780MA device (Tanita, Tokyo, Japan) was used to assess weight and body composition parameters through bioelectrical impedance analysis, including body fat percentage (PBF, %), body fat mass (MBF, kg), lean body mass (LBM, kg), total body water (TBW, kg), and visceral fat level (VFL). Body Mass Index (BMI, kg/m^2^) was calculated by dividing body weight in kilograms by the square of height in meters. Circumferences of the waist, hips, thighs, calves, and ankles were measured using a standard measuring tape with an accuracy of 1 cm. The waist-to-hip ratio (WHR) was obtained by dividing the waist circumference by the hip circumference. Leg circumferences were recorded with a measuring tape to a precision of 0.5 cm. Measurements were taken every 4 cm from the ankle up to the groin along the leg. The volume of the leg (in milliliters) was then calculated using the formula for a truncated cone, based on these measurements [22].

### 2.3. Blood Samples

Blood samples were collected after 12 h of fasting in both groups before and after dietary intervention. The samples were stored in polypropylene tubes designed for freezing biological material at −80 °C. The parameters analyzed included serum IL-6 and hs-CRP concentration.

### 2.4. Serum IL-6 and hs-CRP Determination

Serum concentrations of IL-6 were determined using a commercially available ELISA kit (Diaclone, Codolet, France, Cat. No. 950.030.096), based on a sandwich immunoassay with a solid-phase-bound capture antibody. High-sensitivity CRP (hs-CRP) levels were determined using an hs-CRP assay kit (DiaSys, Holzheim, Germany), based on an immunoturbidimetric method. The assay involved the addition of microparticles coated with anti-human CRP antibodies to buffered serum samples, resulting in immunoprecipitation measured turbidimetrically at 540 nm.

### 2.5. Dietary Intake Assessment of Baseline Diets

Participants’ usual dietary intake over the past year was evaluated using a Food Frequency Questionnaire (FFQ) specifically designed and validated for the Polish population [23]. The questionnaire assessed how often selected foods were consumed, offering 10 frequency options: never, less than once a month, 1–3 times a month, once a week, 2–4 times a week, 5–6 times a week, once daily, 2–3 times daily, 4–5 times daily, and more than 6 times per day. This culturally and regionally tailored FFQ captured average food consumption over the previous year and included 154 food items, which were categorized into 27 food groups. Nutritional intake was calculated using Poland’s national food composition tables [24], with data processed using the Food Processor software (ESHA Research, version 11.x). Portion sizes were estimated based on the ‘Album of Photographs of Food Products and Dishes’ published by the National Food and Nutrition Institute in Warsaw [25]. Nutritional data were obtained from 21 patients in the lipedema group and 24 patients in the overweight/obesity group.

### 2.6. Dietary Intervention

The conventional ketogenic diet primarily emphasizes fat as the main energy source, with little consideration for fat quality. As a result, it often contains a high proportion of saturated fats (e.g., lard, butter, cream, pork, offal, and beef) and relatively low in vegetables. This version is typically low in foods with anti-inflammatory properties. By contrast, the Mediterranean-style ketogenic diet also relies on fat as the principal fuel source but emphasizes healthier fat sources such as plant oils, nuts, seeds, and oily fish, along with lean proteins including skinless poultry, veal, and lean beef. It is further distinguished by a higher intake of vegetables, berry fruits, herbs, spices, and tea, which provide bioactive compounds with anti-inflammatory effects. Thus, the Mediterranean-style ketogenic diet combines the metabolic advantages of ketosis with the well-documented anti-inflammatory benefits of the Mediterranean dietary pattern.

The interventional diet followed a structure similar to a standard ketogenic diet, providing fewer than 50 g of carbohydrates per day (<10% of total daily energy). It was designed in a Mediterranean style, emphasizing the quality of foods consumed and selecting many ingredients for their anti-inflammatory properties. Daily energy intake was divided into three meals, each including a source of protein, fat, and vegetables. Protein sources included eggs, cheeses, lean poultry, and beef, while excluding fatty animal products like pork, offal, and fatty poultry skin. Fat sources focused on foods rich in monounsaturated fatty acids (MUFA), such as olive oil and avocado, as well as polyunsaturated fatty acids (PUFA) from canola and flaxseed oil, nuts and seeds, and fatty fish including salmon, herring, mackerel, and sardines. Each meal also incorporated non-starchy vegetables, including tomatoes, cucumbers, peppers, spinach, sprouts, cauliflower, zucchini, cabbage, radishes, and small servings of berry fruits. The diet was further enriched with herbs and spices known for their anti-inflammatory effects, such as turmeric, cloves, garlic, thyme, rosemary, and included black or green tea once daily. In some cases, a daily snack of nuts, seeds, or fresh vegetables was also added.

The individual weekly 7-day meal plans, which included detailed meal schedules, recipes, and shopping lists were prepared by a clinical dietician using DietetykPro software (https://dietetykpro.pl/, January 2021 to May 2022) (DietetykPro, Wrocław, Poland). These meal plans were followed cyclically over the 7-month intervention period. Each diet plan was individually customized to enhance adherence. Daily energy requirements were calculated individually for each participant based on their resting metabolic rate (RMR) and physical activity level (PAL). RMR was measured with indirect calorimetry using a Fitmate device (Cosmed, Rome, Italy) according to standard protocols [26]. PAL was determined for each participant and generally ranged from 1.3 to 1.5 [27]. Energy intake was then set at approximately 70–90% of each participant’s total energy needs, depending on their body weight. The energy and nutrient content of the intervention diets was calculated using the Polish Tables of Nutritional Value of Products and Dishes [24] as well as the United States Department of Agriculture (USDA) food database [28], with nutrient analysis performed using the same software as described above. A 24 h dietary recall interview was conducted monthly, either by phone or in person, to monitor adherence. Additional assessments were carried out at 2.5 and 5 months during the intervention. These measures helped identify common dietary mistakes, such as consuming high-carbohydrate foods, skipping meals, or restricting fats unnecessarily. Participants who failed to follow the prescribed diet were excluded from the study to maintain the consistency and integrity of the intervention across the study group.

### 2.7. Dietary Inflammatory Index Assessment

For each participant, the Dietary Inflammatory Index (DII) was calculated based on collected dietary intake data for both the pre-intervention period and during the dietary intervention. Nutrient intake values used to estimate the DII components were derived from analyses conducted using the Food Processor software. To estimate the intake of additional bioactive compounds included in the DII—such as flavonoids and isoflavonoids—data from USDA databases on the content of these compounds in selected food items were used.

Based on the available data, it was possible to estimate 43 out of the 45 dietary parameters that comprise the DII, as originally defined by its developers (Table 1) [21]. Two components—trans fatty acids and selenium—were excluded due to the absence of reliable data on their content in available nutritional databases. For each of the 43 parameters included in the DII calculation, the mean daily intake was determined. These values were then standardized based on global reference data by subtracting the global mean intake and dividing by the corresponding global standard deviation, yielding a Z-score for each dietary component. These Z-scores were converted into centered percentile scores, adjusted to range from −1 (most anti-inflammatory) to +1 (most pro-inflammatory) by doubling the percentile score and subtracting one. Each centered percentile was then multiplied by a specific inflammatory effect score assigned to each dietary component. The total DII score was obtained by summing these weighted values. A higher DII score indicates a more pro-inflammatory diet, whereas a lower score reflects a more anti-inflammatory diet.

### 2.8. Statistical Analysis

The Shapiro–Wilk test was used to assess the normality of distribution. To compare the differences between baseline and final anthropometric measurements three types of tests were used: paired Student’s test, Wilcoxon, and Mann–Whitney U-tests (depending on the results of the test checking normality of distribution and the test checking the homogeneity of variation). Differences in DII scores and biochemical markers before and after the implementation of the intervention were evaluated using the paired Student’s *t*-test or the Wilcoxon signed-rank test, depending on whether the data followed a normal distribution. Between-group comparisons were performed on change scores (post–pre) using the independent Student’s *t*-test or the Mann–Whitney U test, as appropriate. Correlations between DII and CRP and IL-6 levels before and after dietary interventions were assessed using Pearson’s correlation coefficient for normally distributed data and Spearman’s rank correlation coefficient for non-normally distributed data. A significance level of *p* < 0.05 was considered statistically significant. Statistical analysis was performed using Statistica 13.1 software (StatSoft, Inc., Tulsa, OK, USA).

Post hoc power analysis using G*Power (version 3.1.9.7) indicated that the achieved power was 0.73 for within-group comparisons (paired *t*-test, α = 0.05, d = 0.5) and 0.52 for between-group comparisons (independent-samples *t*-test, α = 0.05, d = 0.5). Thus, the study was adequately powered to detect medium effects in within-group analyses, but underpowered for between-group comparisons. The achieved post hoc power for correlation analyses ranged from 0.52 (r ≈ 0.41) to 0.83 (r ≈ 0.55).

## 3. Results

### 3.1. Characteristics of Study Group

The median age in the lipedema group was 39.0 years (34.0, 62.0), and 49.0 years (41.5, 59.0) in the overweight/obesity group, with no statistically significant difference between the groups (mean age: 44.5 ± 14.8 years in lipedema, and 50.8 ± 13.3 years in overweight/obesity group). The mean BMI was 31.6 ± 6.4 kg/m^2^ in the lipedema group and 34.0 ± 4.6 kg/m^2^ in the overweight/obesity group, also with no statistically significant difference, indicating that the participants were appropriately matched.

### 3.2. Body Composition and Anthropometric Parameters

After seven months of the intervention, both groups showed significant reductions in body weight, body fat, visceral fat, and all measured anthropometric parameters, including leg circumferences. Detailed changes in measurements following the dietary intervention are presented in Table 2.

### 3.3. Dietary Intake Assessment

The median daily energy and nutrients intake was used to calculate the DII (Table 3). In the lipedema group, the median energy intake at baseline was 1513.8 kcal, with macronutrient distribution of 45% carbohydrates, 20% protein, and 35% fat.

The daily energy intake requirements of participants were individually calculated based on resting metabolic rate and adjusted for physical activity. The mean energy intake requirement was 2089.3 ± 280.0 kcal/day (range 1688–2622 kcal/day) in lipedema group, and 2116.3 ± 299.3 kcal/day (range 1527–2617 kcal/day) in overweight/obesity group. Caloric restriction was subsequently implemented for all patients. During the intervention, the median energy intake was 1670.6 kcal, with 6% carbohydrates, 20% protein, and 74% fat. In the overweight/obesity group, the baseline median energy intake was 1592.5 kcal, consisting of 46% carbohydrates, 18% protein, and 36% fat. The intervention diet in this group was composed of 6% carbohydrates, 21% protein, and 71% fat. There were no statistically significant differences between the dietary intake in intervention diets of the two groups [13].

### 3.4. Dietary Inflammatory Index (DII) Assessment

The DII was reported as median and range in two units: per day and per 1000 kcal, with the latter allowing for the elimination of the confounding effect of total energy intake on the analyzed biochemical parameters. In the lipedema group, a statistically significant reduction in DII was observed between baseline and intervention diets when expressed per day. The median DII/day for habitual diets indicated a pro-inflammatory profile (DII = 3.04), while the intervention diet resulted in a reduction of approximately 1.5 points (*p* = 0.008). When expressed per 1000 kcal, the DII values were markedly lower for both baseline (DII = 0.22) and intervention diets (DII = ~0.01). However, the absolute difference between the two (approximately −0.21) was not statistically significant. In the overweight/obesity group, baseline DII values for habitual diets were 4.00 per day and 1.17 per 1000 kcal. Following the intervention, a statistically significant reduction in DII was observed in both measures, with post-intervention values of 1.27 per day and −0.68 per 1000 kcal. The range of DII values was narrower in the intervention diets compared to the baseline in both groups, which reflects the standardized design of the intervention diets, adjusted for individual preferences. The calculated DII values of patients’ baseline and intervention diets are presented in Table 4.

**Figure 1:**
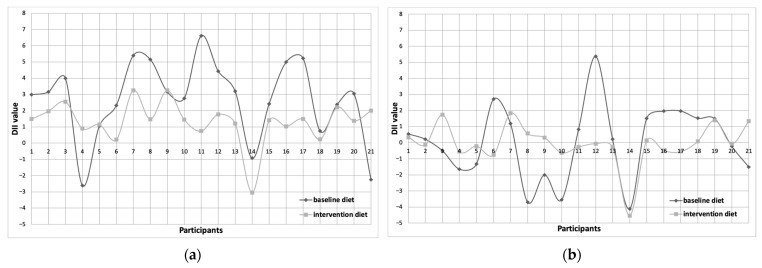
DII value of each lipedema patients at baseline and intervention diets ((**a**)—DII/day; (**b**)—DII/1000 kcal).

**Figure 2:**
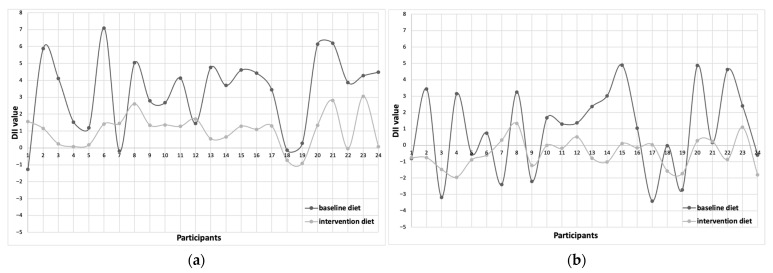
DII value of each overweight/obesity patients at baseline and intervention diets ((**a**)—DII/day; (**b**)—DII/1000 kcal).

Figure 1 and Figure 2 illustrate the individual changes in DII values in baseline and intervention diets. A lower DII/day was observed in the intervention diet compared to the baseline diet in 17 patients with lipedema (Figure 1a). Similarly, when adjusted per 1000 kcal, a reduction in DII was noted in 12 participants (Figure 1b). The largest individual reduction in DII/day was 5.86, while the maximum difference in DII/1000 kcal reached 5.43 (these changes occurred in different individuals). A greater number of participants with overweight/obesity achieved negative DII scores with the intervention diet compared to their baseline diet (Figure 2). A reduction in DII/day was observed in 21 participants, and in 17 participants for DII per 1000 kcal. An increase in DII following the intervention was observed only among participants whose habitual diets had the lowest initial DII values, both per day and per 1000 kcal.

### 3.5. Systemic Inflammation Markers

Following the intervention diet, reductions in CRP and IL-6 levels were observed in both groups. Although the decreases were slightly smaller in the lipedema group for both inflammatory markers, the differences between the groups were not statistically significant (Table 5).

Correlation analysis between the DII scores and the levels of CRP and IL-6 was conducted within each group. In the lipedema group, we noticed significant positive association between DII of intervention diet and CRP level after dietary treatment; similarly in the overweight/obesity group. A significant positive correlation was also observed between baseline DII and IL-6 levels in the lipedema group, but not in the overweight/obesity group (Table 6).

## 4. Discussion

Ketogenic diet is a promising dietary intervention in lipedema treatment. According to recent studies and clinical observations, this dietary approach leads to effective weight loss and fat mass reduction, notably in the lower extremities [11,13]. The ketogenic diet has also shown potential for reducing inflammation [11,29]. The anti-inflammatory properties of the ketogenic diet are also believed to help alleviate symptoms of lipedema by reducing pain and decreasing swelling in the affected areas. Part of its anti-inflammatory effect may be attributed to its role in weight reduction. Elevated BMI and obesity are known to be linked with chronic low-grade inflammation and increased levels of circulating inflammatory markers. Consequently, weight loss can lead to decreased concentrations of cytokines such as CRP, IL-6, and TNF-α [29]. Therefore, assessing inflammation status is crucial, because it may adversely affect the condition of diseased adipose tissue in patients with lipedema [30].

Recommended food products in the ketogenic diet do not align with the typical anti-inflammatory dietary model due to the restriction of many fruits and vegetables, which are key sources of antioxidant compounds such as vitamins—primarily vitamin C and β-carotene. These products also provide polyphenols which exert potent anti-inflammatory effects [31]. The ketogenic diet is high in fat which is considered a pro-inflammatory dietary component, particularly saturated fatty acids and trans fats [32,33]. However, it has already been observed that a calorie-restricted diet emphasizing foods high in anti-inflammatory and antioxidant nutrients may support the overall well-being of individuals with lipedema [9,18,34].

We aimed in this study to evaluate the anti-inflammatory potential of the 7-month of Mediterranean-style ketogenic diet in lipedema patients and assess the changes in systemic inflammation markers. To evaluate the inflammatory potential of the intervention diet, the widely used DII was employed and compared to its values in the participants’ habitual diets [21]. The intervention diet was based on animal-derived products such as lean meat, eggs, and full-fat dairy, which are sources not only of protein but also of saturated fatty acids, known for their pro-inflammatory properties. However, the diet was also rich in plant oils and fish, which provide mono- and polyunsaturated fatty acids—lipids that exhibit anti-inflammatory effects in contrast to saturated fats [35,36]. Furthermore, the intervention diet was designed to closely resemble a Mediterranean-style dietary pattern, which is widely recognized for its anti-inflammatory potential. Therefore, meals were carefully structured to include appropriate amounts of non-starchy vegetables, plant oils, nuts and seeds, berry fruits, herbs, tea and natural coffee [37,38].

It is worth noting that the participants’ baseline diets already reflected attempts to reduce caloric intake, as the median energy value of these diets was lower than the recommended intake for a healthy population. Consequently, although the intervention diet was intentionally energy-restricted to ensure a negative energy balance, its caloric content did not differ substantially from that of the habitual diet [13]. This suggests that the effects of the intervention diet were likely due primarily to its altered macronutrient composition and the increased inclusion of foods rich in anti-inflammatory and antioxidant compounds rather than to differences in energy intake alone. Despite the high fat content, these dietary modifications significantly reduced the median DII score in the intervention diet. It is important to highlight that among the DII components contributing to inflammation, dietary fat carries a relatively high individual DII value of 0.298, compared to 0.021 and 0.097 for protein and carbohydrates, respectively [21]. Thus, it can be inferred that the low inflammatory potential of the intervention diet was primarily attributable to an increased intake of anti-inflammatory compounds that counterbalanced the effects of amount of dietary fat. Moreover, the fat composition of the intervention diet may have also contributed to a favorable modulation of inflammatory status, as previous studies have demonstrated that replacing saturated fatty acids (SFA) with polyunsaturated fatty acids (PUFA)—rather than reducing total fat intake—is associated with reduced systemic inflammation and lower cardiovascular risk [39,40]. In 4 of the 21 participants in the lipedema group, the DII value of the intervention diet increased slightly, which may be attributable to a relatively low inflammatory potential of their habitual diets, leaving limited room for further improvement through dietary modification. Interestingly, no significant change in DII was observed when values were adjusted for energy intake (per 1000 kcal), despite a clear reduction in DII expressed per day. Given the similar total energy content of both dietary patterns, this discrepancy may reflect the specific macronutrient profile of the intervention diet. Although the overall intake of anti-inflammatory components increased, the higher contribution of total dietary fat—an element with pro-inflammatory properties—may have influenced the relative inflammatory density per 1000 kcal. Consequently, the anti-inflammatory effect was more evident in absolute daily intake than when normalized for energy.

To analyze changes in systemic inflammation, we assessed the pro-inflammatory markers hs-CRP and IL-6 and observed that their concentrations significantly decreased following the ketogenic diet. The results of our study are consistent with those reported by Lundanes et al. [9], who demonstrated a significant reduction in CRP levels (−1.4 mg/L) after a 9-week low-carbohydrate diet in patients with lipedema. Similar findings were observed in a study by Cannataro et al. [18], which reported a CRP reduction of 0.4 mg/dL after 7 months on a ketogenic diet, and 0.5 mg/dL after 21 months, in a case report of a lipedema patient. In our study, we also assessed correlations between systemic inflammation markers and the DII, and a positive association was found between CRP concentration and the DII of the ketogenic diet. Interestingly, no association with IL-6 concentration was observed, except for a correlation with DII per day of the baseline diet in the lipedema group. IL-6 is a pro-inflammatory cytokine that stimulates to produce CRP. Elevated levels of IL-6 and CRP in the blood indicate activation of the inflammatory response and are commonly observed in chronic diseases, as well as in lipedema [9]. A decrease in the activity of both markers in the serum may indicate a reduction in the systematic inflammation. In this context, the decrease in serum CRP and IL-6 observed after the intervention suggests that the diet provided compounds that support anti-inflammatory potential. This includes dietary components such as antioxidant vitamins: C, A, and E, unsaturated fatty acids, as well as polyphenolic compounds like quercetin and anthocyanins. However, it should be noted that polyphenols are characterized by low bioavailability and are only minimally absorbed in the small intestine [41]. Nonetheless, they can be metabolized by the gut microbiota in the colon and, in the form of often more biologically active metabolites, exert modulatory effects on inflammation [42].

Targeted dietary approaches, including ketogenic and modified Mediterranean diet, have shown potential in supporting the management of lipedema by lowering inflammation and enhancing metabolic health. Developing personalized treatment plans that address the specific challenges of lipedema—such as its resistance to standard weight-loss methods and its inflammatory component—is essential for achieving optimal outcomes [29]. The results obtained in this study indicate that a ketogenic diet rich in anti-inflammatory and antioxidant components may have a reduced pro-inflammatory potential, despite its high fat content. Moreover, it does not lead to an increase in systemic inflammation; on the contrary, it contributes to a significant reduction in inflammation, as measured by CRP and IL-6 levels, particularly through a high consumption of bioactive substances found in fishes, plant oils, nuts, vegetables, berry fruits, and spices. Additionally, the observed reduction in inflammation may be partly attributed to weight loss achieved by the participants, including a decrease in adipose tissue. To better understand the mechanisms underlying the effects of the investigated ketogenic diet—especially its impact on systemic inflammation in women with lipedema—further studies involving a larger cohort and comprehensive assessment of inflammatory markers are warranted.

Limitations of the study include the relatively small sample size, which may limit the generalizability of the findings. Baseline dietary intake was derived from self-reported records, which are subject to recall bias and potential under- or over-reporting. Finally, while the 7-month intervention provides valuable medium-term data, longer follow-up is needed to assess the sustainability of dietary adherence and the long-term effects on inflammation. Larger, randomized controlled trials are needed to confirm these findings and improve generalizability. Longer follow-up would help assess dietary adherence and the durability of anti-inflammatory effects. Expanding the biomarker panel and considering additional lifestyle factors could also provide deeper insight into the mechanisms linking diet, inflammation, and lipedema.

## 5. Conclusions

The ketogenic diet designed in a Mediterranean style had a lower Dietary Inflammatory Index compared to the habitual diet of patients with lipedema, despite having a similar energy value. The diet caused a decrease in systemic inflammation markers (CRP and IL-6) indicating a protective effect of the anti-inflammatory and antioxidant components provided by this diet, despite its high fat content. The findings suggest that the nutrient composition—rather than caloric restriction or weight loss alone—is responsible for the greater reduction in inflammation observed in the study group. Furthermore, the observed associations between DII and inflammatory markers in the lipedema group, both before and after the intervention, suggest that DII expressed per day may reliably reflect the diet-induced changes in systemic inflammation in this patient population.

## Figures and Tables

**Table 1:** Nutrients and food items included in the DII calculation (43 parameters).

Group	Nutrients and Foods
Nutrients	energy value, protein, carbohydrates, fiber, total fat, saturated fatty acids, cholesterol, monounsaturated fatty acids, polyunsaturated fatty acids, omega-3 fatty acids, omega-6 fatty acids, alcohol, vitamin A and β-carotene, vitamins D, E, thiamine, riboflavin, niacin, vitamins B_6_, B_12_, C, and folic acid, zinc, magnesium, iron
Plant bioactives	anthocyanins, eugenol, flavan-3-ols, flavones, flavonols, flavanones, isoflavones, caffeine
Products with anti-inflammatory properties	onion, garlic, ginger, turmeric, pepper, rosemary, saffron, thyme + oregano black + green tea

**Table 2:** Body composition and anthropometric parameters before and after dietary intervention in both study groups.

Parameter	Lipedema Group (n = 24) Mean ± SD/Me (Q1, Q3)			Overweight/Obesity Group (n = 24) Mean ± SD/Me (Q1, Q3)		
	Before	After	*p*-Values	Before	After	*p*-Values
Weight [kg]	86.1 ± 17.8	74.1 ± 12.9	<0.0001	90.5 (81.7, 97.5)	77.1 (69.2, 86.5)	<0.0001
BMI [kg/m^2^]	31.6 ± 6.4	27.3 ± 5.1	<0.0001	33.9 (30.5, 36.3)	28.0 (26.1, 33.1)	<0.0001
PBF [%]	39.9 (35.7, 42.8)	33.8 (27.8, 37.4)	<0.001	40.5 (36.8, 43.6)	33.2 (28.9, 39.3)	<0.001
MBF [kg]	33.6 (28.4, 39.2)	25.2 (18.8, 30.2)	<0.001	36.7 (29.3, 43.1)	25.5 (20.0, 34.5)	<0.001
VFL	14.0 (8.5, 16.5)	9.5 (4.0, 12.0)	<0.001	10.5 (8.0, 13.5)	8.0 (6.0, 10.0)	<0.001
Waist [cm]	98.0 ± 12.9	85.2 ± 11.3	<0.0001	106.7 ± 10.6	94.4 ± 12.1	<0.0001
Hips [cm]	115.1 ± 12.0	105.4 ± 9.2	<0.0001	115.0 (109.5, 119.8)	104.8 (101.0, 108.3)	<0.0001
WHR	0.84 (0.78, 0.91)	0.79 (0.74, 0.86)	0.0001	0.93 ± 0.1	0.9 ± 0.1	0.006
Left thigh [cm]	65.1 ± 7.5	59.1 ± 5.9	<0.0001	64.9 ± 5.6	59.2 ± 5.3	<0.0001
Right thigh [cm]	65.1 ± 7.3	58.9 ± 6.0	<0.0001	64.9 ± 5.8	59.1 ± 5.4	<0.0001
Left calf [cm]	44.7 ± 5.2	40.7 ± 3.9	<0.0001	42.1 ± 3.9	39.5 ± 3.7	<0.0001
Right calf [cm]	44.5 ± 5.3	40.8 ± 4.3	<0.0001	42.4 ± 4.1	39.9 ± 4.0	<0.000
Left ankle [cm]	25.0 ± 2.3	23.7 ± 2.1	<0.0001	23.0 (22.3, 24.8)	23.0 (22.3, 24.5)	NS
Right ankle [cm]	24.0 (23.0, 26.0)	23.0 (22.3, 24.8)	0.0001	23.9 ± 2.5	23.7 ± 2.4	NS

BMI—body mass index, PBF—percentage body fat, MBF—mass of body fat, VFL—visceral fat level, WHR—waist–hip ratio.

**Table 3:** Characteristics of energy and nutrients content at baseline and intervention diet.

Parameter	Lipedema (n = 21)		Overweight/Obesity (n = 24)	
	Baseline Diet Me (Q1, Q3)	Intervention Diet Me (Q1, Q3)	Baseline Diet Me (Q1, Q3)	Intervention Diet Me (Q1, Q3)
Energy [kcal]	1513.8 (641.5–2421.0)	1670.6 (1424.7–2022.0)	1592.5 (626.8–2515.9)	1687.5 (1401.0–1866.0)
Total protein [g]	74.1 (34.0–153.9)	86.7 (65.1–113.4)	69.9 (30.4–109.1)	88.9 (79.9–102.2)
Total carbohydrates [g]	175.5 (20.0–346.7)	29.6 (23.1–85.0)	182.4 (68.2–310.6)	30.4 (24.8–37.7)
Fiber [g]	17.2 (2.5–31.2)	8.6 (6.7–24.5)	14.9 (5.8–24.1)	8.7 (6.7–20.6)
Fat [g]	58.7 (32.0–119.4)	136.6 (102.4–154.2)	63.0 (26–95.4)	133.4 (70.6–147.2)
SFA [g]	22.8 (8.2–50.4)	38.1 (23.8–52.3)	25.3 (8.5–38.4)	36.8 (22.7–52.3)
MUFA [g]	22.2 (9.6–44.4)	58.0 (38.0–69.1)	23.2 (9.6–37.2)	56.3 (44.0–78.5)
PUFA [g]	9.5 (5.9–16.5)	25.0 (14.8–39.6)	10.7 (3.1–15.2)	26.5 (12.9–47.1)
Total *n*-3 [g]	1.4 (0.6–3.5)	4.0 (0.8–8.7)	1.5 (0.6–2.4)	4.6 (2.5–5.8)
Total *n*-6 [g]	7.8 (3.8–13)	10.2 (7.9–17.6)	8.8 (2.5–13.3)	12.4 (7.5–20.6)
Cholesterol [mg]	261.8 (97.8–728.2)	608.7 (469.4–798.8)	288.3 (183.7–655.6)	622.1 (301.6–801.4)
Thiamine [mg]	1.0 (0.5–1.7)	0.8 (0.5–6.3)	0.9 (0.3–1.6)	0.8 (0.5–1.2)
Riboflavin [mg]	1.5 (0.7–2.9)	2.1 (1.3–4.1)	1.6 (0.6–2.6)	1.9 (1.3–4.8)
Niacin [mg]	19.2 (6.0–41.7)	18.8 (13.2–27.1)	19.3 (9.1–30.2)	18.7 (9.2–31.1)
Vitamin B6 [mg]	1.6 (0.6–3.2)	1.7 (1.1–2.7)	1.5 (0.6–2.2)	1.9 (1.4–3.4)
Folate [µg]	252.8 (148.6–486.6)	327.6 (217.1–479.7)	243.0 (129.0–383.7)	320.9 (190.0–416.0)
Vitamin B12 [µg]	3.3 (0.7–8.3)	8.0 (4.1–17.0)	3.5 (1.0–6.4)	6.7 (4.2–21.2)
Vitamin C [mg]	97 (31.1–284.4)	118.6 (83.1–220.2)	107.8 (31.8–204.6)	128.5 (71.1–205.8)
Vitamin A [µg]	1108.3 (528.6–3130.1)	1234.3 (699.8–1954.4)	1035.5 (583.4–2190.3)	1178.1 (673.1–3657.1)
β-Carotene [µg]	3609.0 (1082.8–8504.9)	3151.3 (1868.6–7696)	2935.4 (1822.8–8947.5)	3492.0 (2016.9–6820.3)
Vitamin D [µg]	2.0 (0.5–5.5)	9.1 (4.5–18.7)	1.9 (1.2–3.6)	8.5 (5–10.8)
Vitamin E [mg]	8.9 (5.7–17.3)	16.4 (13.4–28.7)	9.6 (3.7–14.0)	19.7 (11.9–30.0)
Zinc [mg]	9.2 (4.6–15.8)	8.4 (6.6–12.1)	8.3 (3.6–13.5)	9.1 (6.5–17.4)
Magnesium [mg]	314.9 (126.4–579.7)	223.4 (153.6–766.9)	325.4 (94.8–493.3)	259.7 (178.8–414.6)
Manganese [mg]	5.1 (2.4–9.0)	1.2 (0.7–5.4)	4.2 (2.2–8.1)	1.5 (0.7–2.6)
Copper [mg]	1.3 (0.5–2.4)	0.9 (0.7–1.6)	1.3 (0.3–1.8)	1.0 (0.7–1.5)
Iron [mg]	10.4 (6.0–17.6)	9.7 (7.3–15)	10.7 (4.6–14.7)	9.2 (6.5–13.0)

SFA—Saturated fatty acids; MUFA—Monounsaturated fatty acids; PUFA—Polyunsaturated fatty acids; *n*-3—omega-3 fatty acids, *n*-6—omega-6 fatty acids.

**Table 4:** DII values of the participants’ baseline and interventional diets.

	Lipedema (n = 21)			Overweight/Obesity (n = 24)			Differences Between Before and After Intervention		
	Baseline Diet Me (Q1, Q3)	Interventional Diet Me (Q1, Q3)	*p*-Value	Baseline Diet Me (Q1, Q3)	Interventional Diet Me (Q1, Q3)	*p*-Value	Lipedema	Overweight/Obesity	*p*-Value
DII/day	3.04 (−2.60–6.61)	1.45 (−3.0–3.26)	0.008	4.00 (−1.27–7.09)	1.27 (−0.91–3.05)	<0.001	−1.50 (−2.13–−0.48)	−2.65 (−3.89–−1.19)	NS
DII/1000 kcal	0.22 (−4.12–5.37)	−0.06 (−4.54–1.83)	NS	1.17 (−3.41–4.89)	−0.68 (−1.95–1.33)	<0.001	−0.21 (−5.43–4.27)	−1.32 (−5.50–3.44)	0.044

DII—Dietary Inflammatory Index.

**Table 5:** Markers of systemic inflammation before and after dietary intervention.

Parameter	Lipedema Group Me (Q1, Q3)			Overweight/Obesity Group Me (Q1, Q3)			Differences Between Before and After Intervention		
	Before Me (Q1, Q3)	After Me (Q1, Q3)	*p*-Value	Before Me (Q1, Q3)	After Me (Q1, Q3)	*p*-Value	Lipedema Me (Q1, Q3)	Overweight/Obesity Me (Q1, Q3)	*p*-Value
hs-CRP [mg/dL]	3.44 (2.00–5.55)	2.99 (1.84–4.73)	0.016	3.76 (3.22–4.37)	2.46 (2.60–4.02)	0.001	−0.39 (−0.89–0.26)	−0.56 (−1.64–−0.04)	NS
IL-6 [pg/mL]	8.93 (7.22–10.5)	8.54 (6.48–9.42)	0.034	9.11 (7.62–10.6)	7.62 (7.56–9.63)	0.014	−0.33 (−1.38–−0.28)	−0.95 (−1.91–0.13)	NS

hs-CRP—high-sensitivity C-reactive protein; IL-6—interleukin 6.

**Table 6:** Correlation analysis of DII score with CRP and IL-6 levels in the study groups.

DII	Lipedema Group				Overweight/Obesity Group			
	Before/Baseline Diet		After/Intervention Diet		Before/Baseline Diet		After/Intervention Diet	
	hs-CRP	IL-6	hs-CRP	IL-6	hs-CRP	IL-6	hs-CRP	IL-6
DII/day	r = 0.34	r = 0.50	r = 0.55	r = 0.17	r = 0.18	r = 0.32	r = 0.41	r = 0.22
	*p* = 0.106	*p* = 0.013	*p* = 0.005	*p* = 0.440	*p* = 0.393	*p* = 0.131	*p* = 0.044	*p* = 0.297
DII/1000 kcal	r = 0.08	r = 0.27	r = 0.41	r = −0.01	r = −0.07	r = 0.04	r = 0.44	r = 0.39
	*p* = 0.713	*p* = 0.207	*p* = 0.047	*p* = 0.961	*p* = 0.712	*p* = 0.864	*p* = 0.031	*p* = 0.063

hs-CRP—high-sensitivity C-reactive protein; IL-6—interleukin 6; DII—Dietary Inflammatory Index.

## Data Availability

All data used to support the findings of this study are available from the corresponding author upon reasonable request due to the privacy of patients.
